# Comparative Analysis of Sedative Efficacy of Dexmedetomidine and Midazolam in Pediatric Dental Practice: A Systematic Review and Meta-Analysis

**DOI:** 10.7759/cureus.28452

**Published:** 2022-08-26

**Authors:** Ranu R Oza, Varsha Sharma, Tejas Suryawanshi, Saniya Lulla, Pavan Bajaj, Prasad Dhadse

**Affiliations:** 1 Department of Periodontics and Implantology, Sharad Pawar Dental College and Hospital, Datta Meghe Institute of Medical Sciences, Wardha, IND; 2 Department of Pedodontics and Preventive Dentistry, Sharad Pawar Dental College and Hospital, Datta Meghe Institute of Medical Sciences, Wardha, IND

**Keywords:** sedation, pediatric preventive dentistry, systematic review and meta analysis, midazolam, dexmeditomidine

## Abstract

Children are particularly terrified of having dental treatment. They are physically resistant, frail, and unwilling to cooperate. This severe distress during the pre-operative phase could cause the dentist to have issues with behavior control. Additionally, it may make pediatric dental treatments less effective. In order to reduce anxiety and control behavior in children receiving dental care, sedation is a pharmacological management technique that supports the provision of effective and high-quality dental services. The aim is to compare and evaluate the efficacy of sedative agents like dexmedetomidine and midazolam in pediatric dental practice. A thorough review of the literature was conducted using electronic databases like “MEDLINE, PubMed, and CENTRAL (Cochrane Central Register of Controlled Trials), as well as the World Health Organization International Clinical Trials Registry Platform, www.clinicaltrials.gov, conference proceedings abstracts, a bibliography of pertinent references, and manual searches of journals, conferences, and books”. There were no restrictions on the language or the date of publication when searching the electronic databases.

Randomized controlled trials were included which compared dexmedetomidine and midazolam in children up to 16 years of age subjected to dental treatment. Information on procedures, participants, interventions, outcome measures, and results were independently extracted by three review writers (TS, SL, and RO). Trial authors were contacted for papers that were confusing or lacking information. The risk of bias was evaluated for each study. We adhered to the Cochrane statistical recommendations. Three trials totaling 229 participants were included. All three studies were rated as having a low risk of bias, with none of them having a high or unclear risk. Meta-analysis was done for the available data for the primary outcomes like sedation level and recovery time. We searched for randomized controlled trials up to Jan 31, 2020. Participants are randomly assigned to an intervention or control group in randomized controlled trial research. While patients in the control group often get a placebo therapy or procedure, those in the interventional group receive the treatment being studied.

We found three studies eligible to include in the review. One study evaluated 73 individuals who received general anesthesia for dental treatment. There were 72 and 84 individuals in the second and third investigations, respectively. All the participants of the three studies were divided randomly into two groups and were subjected to dexmedetomidine and midazolam as sedative agents. We gave the evidence an "extremely low certainty" rating. Because there are just three short trials with unusual parameters for comparison, the results are questionable. Overall, the results do not allow us to draw any firm conclusions. Three randomized controlled trials included in this systematic review reported data with varying conclusions; hence we recommend more randomized controlled trials to be conducted on this subject matter.

## Introduction and background

Description of the condition

The pediatric population is one of the more susceptible groups of patients. Treating them can be difficult because of the various emotional factors involved, such as pain, fear of the unknown, and rage. The therapy options available to the treating pedodontist include behavior control in a big way. In order to calm the child's tension and anxiety and make him or her amenable to behavior management, pharmacological techniques like sedation are used [1]. Due to its anxiolytic, sedative, hypnotic, and amnesic effects, midazolam is the most popular premedication of the several pharmacological substances that have been utilized. Extensive research has been done on the use of dexmedetomidine, a recent addition to a family of beta 2 agonists, in the pediatric population as an adjuvant sedative and local anesthetic in children [2].

How the intervention might work

The goal of sedation is to lower a patient's level of CNS activity by administering one or more medicines in various combinations. This lowers the patient's level of awareness of their surroundings [3]. The term procedural sedation (PSA) has coined by The American College of Emergency Physicians (ACEP) [4]. Procedural sedation is the process of giving patients sedatives or dissociative drugs along with or without analgesics to create a state that will allow them to endure uncomfortable procedures while still sustaining their heart, lungs, and other vital organs. The goal of PSA is to provide a low level of consciousness that enables the patient to autonomously maintain their oxygenation and control of their airways. Though PSA is widely practiced and a time-tested procedure for minor procedures, it has some inherent risks and drawbacks such as sharing the anesthesiologist's field with that of the dentist and airway co-occurring illnesses including epilepsy, mental instability, and cardiac abnormalities [5]. Trigeminal nerve stimulation during surgery increases the risk of arrhythmias, and children with enlarged tonsils and adenoids are more likely to develop respiratory obstruction [6]. Other complications may include the risk of respiratory and cardiovascular depression, and vasovagal syncope [7]. Hence, although conscious sedation might look safe, logistical, and economical option, the complications arising from the procedure might be difficult to manage in a regular dental-clinical setup. So, depending on the nature of the case, the choice between general anesthesia and sedation is crucial [8].

Over the past few decades, there has been an exponential increase in the number of minimally invasive and noninvasive operations performed outside of the operating room [7]. For several of these interventional or diagnostic procedures, analgesia, sedation, or both may be necessary (such as computed tomography, magnetic resonance imaging or mask placement, and intra-venous line establishment) [9]. A sizable portion of patient preparation and pre-operative patient management is made easier by the development of shorter-acting sedative drugs for sedation, newer effective and selective opioids for effective pain control, and particular reversal medicines for benzodiazepines [10].

Why is it important to do this review?

In the era of evidence-based medical practice, adequate supporting studies are needed at every procedural step. At the same time, newer drugs are coming up into practice and challenging the previously stated gold standards, making it difficult for a clinician to decide on the drug of choice [11]. One of the traditional sedatives for PSA is midazolam. Although midazolam is expected to have only minor hemodynamic effects, it has the potential to weaken or lose airway reflexes, depress breathing, and also cause apnea [12]. If an efficiently, effective, and safe sedative could be used in routine general practice, this would benefit a wide range of patients, especially those who are uncooperative, and will also aid the performing dental surgery in the procedure [13].

## Review

Objective

To compare and evaluate dexmedetomidine and midazolam as sedative agents in dental practice for children.

Methods

We included randomized controlled trials (RCT) evaluating the effectiveness of dexmedetomidine against midazolam in the pediatric population (children of age group 3-18 years) subjected exclusively to dental treatment with blinded evaluation of outcomes. We necessitated complete journal publication with the inclusion of extended abstracts of unpublished clinical articles. We also excluded the non-randomized studies, animal research studies, case-control studies, clinical prospective observational studies, and case reports and discussions. Regardless of sex, color, social class, or economic standing, we examined trials with individuals who matched the criteria of the population that targeted pediatric subjects only. Studies with subjects indicated exclusively for dental treatment were included. Study designs pertaining to randomized controlled trials were included. Types of intervention: Experimental- dexmedetomidine used in any concentration by any suitable mode of delivery and route of administration was preferred; Comparison- midazolam used in any concentration by any suitable mode of delivery and route of administration was preferred.

Types of outcome measures were mean onset of sedation- measured to determine how long it takes for the effects of the medicine to effectively peak after delivery, the mean onset of sedation is the time between taking the premedication and becoming sedated; mean level of sedation- the depth of sedation patients goes into in the post-intervention period is measured pre-anesthetically; mean time of recovery- the amount of time it took to score a 9 on the modified Aldrete recovery scale, which was used to measure how well people recovered from anesthesia reversal. Search methods included electronic searches. We carried out electronic searches from (from inception till February 2019) on the following platforms: MEDLINE, PubMed, CENTRAL, Cochrane, and www.clinicaltrials.gov. We excluded Embase because those articles we got from the Cochrane database. An electronic search strategy was performed using the following keywords- dexmedetomidine, midazolam, pediatric dental practice, and pediatric anesthesia. Search strategy used was: ("dexmedetomidine"{Title/Abstract} OR "dexmedetomidine"{MeSH Terms}) AND ("midazolam"{MeSH Terms} OR "midazolam"{Title/Abstract}) AND ("pediatric dentistry"{Title/Abstract} OR "pediatric dentistry"{MeSH Terms}). Other sources were also checked like various conference proceedings and abstracts and other searched bibliographies for the relevant references, manual searches for journals, conference abstracts, and books contacting experts in the field for any trials with the potential for eligibility. Hand searching included pertinent information, we manually searched all of the English professional journals. Additionally, we made sure that none of the reviews' included research had ever been retracted for fraud or error. We only took into account unfavorable effects that were mentioned in included studies; we did not conduct a separate search for treatments' negative impacts.

Study selection

The following inclusion selection criteria were applied to the population: children undergoing dental surgery; intervention: dexmedetomidine; comparison: midazolam and study design: RCTs.

Data management

Three independent reviewers (TS, SL, RO) extracted data from included studies using a predefined data extraction form, and the data is presented in the Characteristics of Studies table [14]. Data were extracted in terms of the type of study, details of participants, details of the intervention, and the outcomes reported. The fourth reviewer (PB) resolved the discrepancy amongst the primary reviewers. The discrepancy in the risk of bias assessment was resolved by the fifth reviewer (PD). When necessary, the corresponding authors were contacted to obtain the data required. There are no simplifications or assumptions made [15].

Assessment of risk of bias

Three reviewers (SL, TS, and VS) independently assessed the risk of bias (ROB) of each included study using the Cochrane domain-based, two-part tool as described in chapter 8 of the Cochrane handbook for systematic reviews of interventions (Higgins, 2011) [16]. The discrepancy among the primary reviewers was resolved by the fourth reviewer (PB). We assessed the ROB essentially under the following seven domains: (1) Random sequence generation- selection bias (biased allocation to interventions) due to inadequate generation of a randomized sequence; (2) Allocation concealment- selection bias (biased allocation to interventions) due to inadequate concealment of the allocation; (3) Blinding of participants and personnel- performance bias due to knowledge of the allocated interventions by participants and personnel during the study; (4) Blinding of outcome assessment- detection bias due to knowledge of the allocated interventions by outcome assessors; (5) Incomplete outcome data- attrition bias due to amount, nature, or handling of incomplete outcome data; (6) Selective reporting: reporting bias due to selective outcome reporting; (7) Other bias: bias due to problems not covered elsewhere in the table, such as baseline imbalance, confounding, contamination, co-interventions, etc. [17].

Measures of treatment effects

Meta-analysis was done only after confirming that subjects, interventions, comparisons, and outcomes were found to be sufficiently similar. Review manager 2014, the statistical package provided by the Cochrane collaboration for meta-analysis, was employed [18]. Only when it was determined that the data was sufficiently homogeneous with comparable results was meta-analysis performed using a fixed-effect model. We sought to find the origins of statistical heterogeneity (I^2^ 70%), probed into the causes of heterogeneity using the subgroup analysis, and then did a meta-analysis using a random-effects model [19]. Since the meta-analysis looked appropriate, we conglomerated the results of the included studies; rather than portraying a qualitative description of these studies with supporting data [20].

Issues with the unit of analysis

In contrast to RCTs, we intended to assess cluster-randomized trials and studies with more than two intervention groups. Following the recommendations of the first edition of the Cochrane handbook for systematic reviews of interventions, we would have utilized approximate analysis of selected sample sizes for cluster-randomized trials to prevent any inadvertent analyses in the original studies (Higgins, 2011) [16]. As each meta-analysis only looked at a single pair-wise comparison for papers with more than two intervention groups, we would have taken into account two strategies. If the first strategy failed, we intended to choose the most pertinent pair of interventions. The first was to combine groups to form a single pair-wise comparison [21].

Missing data

For trials that had missing data, we got in touch with the trial authors to get further information and complete the data. In the event that there was no response, we intended to use the following statistical techniques in accordance with the recommendations in the Cochrane handbook for systematic reviews of interventions, first edition [22].

Heterogeneity assessment

To determine whether there was heterogeneity among the included studies, we intended to utilize the Chi^2^ test for heterogeneity [23]. To gauge the effect of the heterogeneity, we employed the I^2^ statistic: Depending on the percentage, small heterogeneity can range from 0% to 40%; moderate heterogeneity can range from 30% to 60%; substantial heterogeneity can range from 50% to 90%, and very large (considerable) heterogeneity can range from 75% to 100% [24].

Reporting biases

We conducted a thorough search that includes grey literature and ongoing studies to prevent reporting biases (see search methods for identification of studies) [25]. If more than ten trials provided findings for one outcome, we intended to use funnel plots to analyze publication bias and other reporting biases. The methods provided by Egger 1997 (continuous outcomes) and Rücker 2008 would have further explored the hypothesis that asymmetry of the funnel plot can indicate potential publication bias (dichotomous outcomes) [26].

Synthesis of data and subgroup analysis

If more than one study used comparable comparisons and reported the same result, we planned to pool the data using a fixed effect meta-analysis model for two to three studies and random effects for four or more studies [27]. We discovered statistical heterogeneity (I^2^ 70%), sought to understand the causes of the heterogeneity by the subgroup analysis, and then conducted a meta-analysis using a random-effects model. Since the meta-analysis looked appropriate, we conglomerated the results of the included studies, rather than portraying a qualitative description of these studies with supporting data [28].

Sensitivity analysis and summary of main results

We did not run any sensitivity tests. Regarding the missing data, we examined only the available data (i.e., ignore the missing data), and assessed how sensitive the results were to reasonable changes in the assumptions that were made. We did this under the assumption that the data were missing at random. In order to deliver key information on the comparison of dexmedetomidine with midazolam as a sedative agent in pediatric dental treatment, we formed a table for each study, comparing them on even grounds. This exhibits the certainty of the evidence for all the outcomes (onset of sedation, postoperative analgesia, and recovery time). The evaluation of the body of evidence included consideration of the risk of bias at the heterogeneity, directness of the evidence, outcome level, and precision of effect estimates.

Results

We included three RCTs in this review [29-31]. See the characteristics of included studies for full details in Table 1.

**Table 1:** Summary of included articles Reference nos. [29-31] RCT: Randomized controlled trials; NR: Not reported; ASA: American Society of Anesthesiology; IN: Intranasal; IV: Intravenous; SpO2: Saturation of peripheral oxygen; RR: Respiratory rate; DBP: Diastolic blood pressure; SBP: Systolic blood pressure; AAPD: American Academy of Pediatric Dentistry; CHEOPS: Children's Hospital of Eastern Ontario Pain Scale

Article	Sheta et al., 2014 [29]	Sathyamoorthy et al., 2019 [30]	Surendar et al., 2014 [31]
Country	United Arab Emirates	United States	India
Type of study	RCT	RCT	RCT
Details of the group			
Number of groups	2	2	4
	Group M: Midazolam	Group M: Midazolam	Group D1 and D2 Dexmeditomedine,
	Group D: Dexmedetomidine	Group D: Dexmedetomidine	Group M1:Midazolam, Group K1: ketamine
Number of patients	72		84
Number of patients after the trial	72	73	84
Sex	NR	Male: 50 Female: 23	Male: 43 Female: 41
Participation details			
Age range	3-6 years	5-18 years	4-14 years
Weight Range	14.6 - 22.3 kgs	20-146 kgs	9-27 kgs
Neurobehavioral Disorder	0	12	0
ASA physical status (I/II)	59/13		84/0
Intervention			
Type	Dexmedetomidine and Midazolam	Dexmeditomedine and Midazolam	Dexmeditomedine ,Ketamine and Midazolam
Dose of Dexmedetomedine	1μg/kg (max dosage-10μg/kg)	2 mcg/kg (max dosage-100μg/kg)	Group D1- 1μg/kg Group D2-1.5μg/kg
Mode of Delivery	IN	IN	IN
Preparation	100mg/ml paraentral preparation + NS = 1 ml syringe	Undiluted paraentral Prep used	Calculated dosage+ NS = 2 ml
			Ketamine 5mg/Kg, Intranasal
Dose of Midazolam	0.2mg/kg (Max dosage- 5mg/kg)	0.5mg/kg (Max dosage-15mg)	5mg/kg
Mode of delivery	IN	Oral	IN
Preparation	5mg/ml paraentral prep.+ 0.9ml saline = 1ml	5mg/3ml premixed flavor syrup with hospital pharmacy	5mg/kg paraentral prep.+ NS = 2 ml
IN Drug Delivery Device	1ml needle less syringe	Intranasal mucosal atomizer device	1ml needleless syringe
Post-Op Analgesics	Rectal Paracetamol (30-40mg/kg),Morphine (25μg/kg)	Morphine 0.05-0.1 mg/kg ,Ketorolac (0.5mg/kg, Max dosage -30 mg)	IV Morphine
IN Drug Volume	2ml in two aliquots	NR	2 ml in two aliquots
Outcomes			
Primary Outcomes	Level of Sedation upon separation from their parents	Level of sedation upon separation from their parents	SpO2, RR, DBP, SBP, pulse rate, onset time, recovery time, intraoperative analgesia, post-operative analgesia
Sedation Scale	4-point scale subject with a score ≥2 was transferred to OR.	5 point Sedation scale (University of Michigan Sedation Scale)	5-point scale modified from AAPD Sedation record
Secondary Outcomes	To study the level of Anxiolysis, Recovery time	To study the level of anxiolysis/child compliance at induction.	NR
Anxiolysis scale	4-point scale (score of 3 or 4 was considered acceptable1 or two was unacceptable)	4 point scale	NR
Recovery Scale	>9 scores on the Modified Aldrete Scale	NR	>9 scores on the Modified Aldrete Scale
Local Anesthesia	Local infiltration (xylocaine 20mg/ml and adrenaline 12.5 mcg/ml)	Not given	Nerve block (2% lignocaine with 1:200000 adr)
Postoperative Pain Scale	Less CHEOPS less no. of rescue analgesics consumed with dexmedetomidine	NR	Intra and postoperative analgesic effect, measured using the Face, Legs, Activity, Cry, Consolability (FLACC) scale.
Adverse Reaction	None	None	Vomiting (1)
Dexmedetomidine	None 36.1% Nasal irritation and Teary eyes	None None	Vomiting (1) None
Adverse reactions with Midazolam			
Definitions: 1) Onset of Sedation	According to sedation scores, the minimum time interval necessary for the child to become drowsy or fall asleep ( ≥2) was considered as the onset of Sedation.	Not Defined	Not Defined
2. Sedation	Not Defined	Not Defined	Safe and successful Sedation- physiological parameters within 10 % of baseline,”

Description of studies

Results of the Search

**Figure 1:**
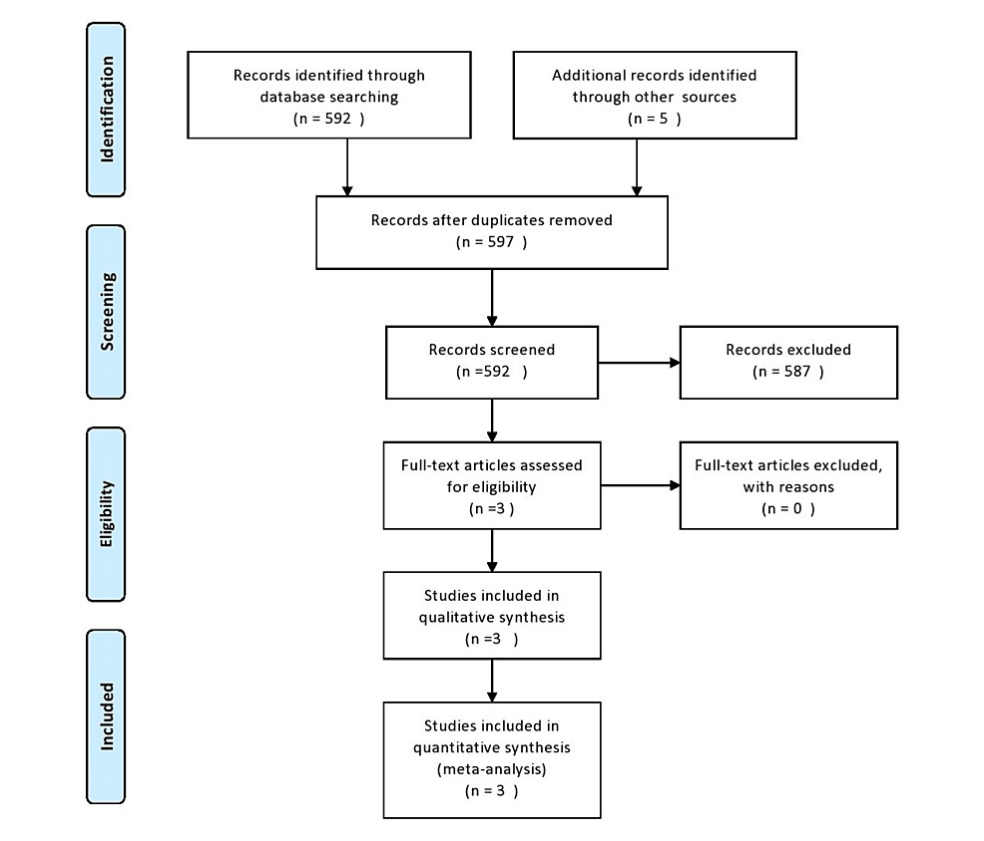
Prisma flow diagram

We identified a total of 597 records from electronic searches and hand searching. After removing duplicates, we screened 592 records by scanning the titles and abstracts. We considered three records to be potentially eligible and obtained the full texts for further consideration. We included three randomized control trials in this review [29-31]. A PRISMA (Preferred Reporting Items for Systematic Reviews and Meta-Analyses) flow diagram (Figure 1) illustrating the study selection process is shown according to the guidelines [32].

Included Studies

Intervention: The included studies evaluated the efficacy of dexmedetomidine and midazolam as sedative agents in pediatric dental practice aged 3-18 years. All the studies randomly divided patients into two groups, and the interventional group was administered dexmedetomidine via the intranasal route in varying doses, while a predefined dose of midazolam was given to subjects in the control group. The dose of dexmedetomidine that was given in the included studies was in the range of 1 mg/kg to 2 mg/kg, and the mode of drug delivery was intranasal. The drug delivery device used was a 1 ml needless syringe, and in the study of intranasal mucosal atomizer device was used. Control: All the included studies had two groups comparing dexmedetomidine and midazolam except one which had a third group that also compared ketamine. In all the studies, midazolam was administered from 0.2mg/kg to 5mg/kg via intranasal and oral routes. The intranasal drug delivery device used was a 1 ml needless syringe in the study of the intranasal mucosal atomizer device was used.

Outcome

Primary outcome: Two of three included trials collected data depending upon the level of sedation, which included intraoperative analgesia, postoperative analgesia, and recovery time, all as its primary outcome. Secondary outcome: Included trials collected data on recovery time and level of anxiolysis.

Risk of bias in the included studies

**Figure 2:**
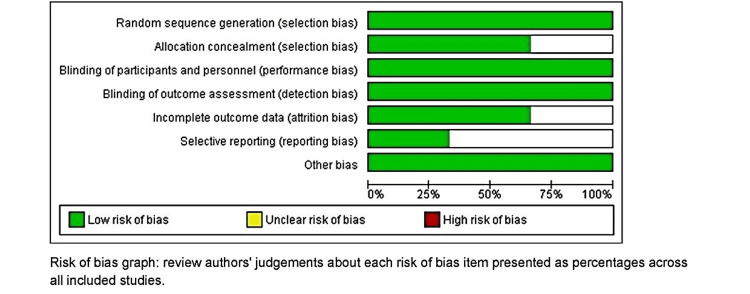
Risk of bias in included studies

**Figure 3:**
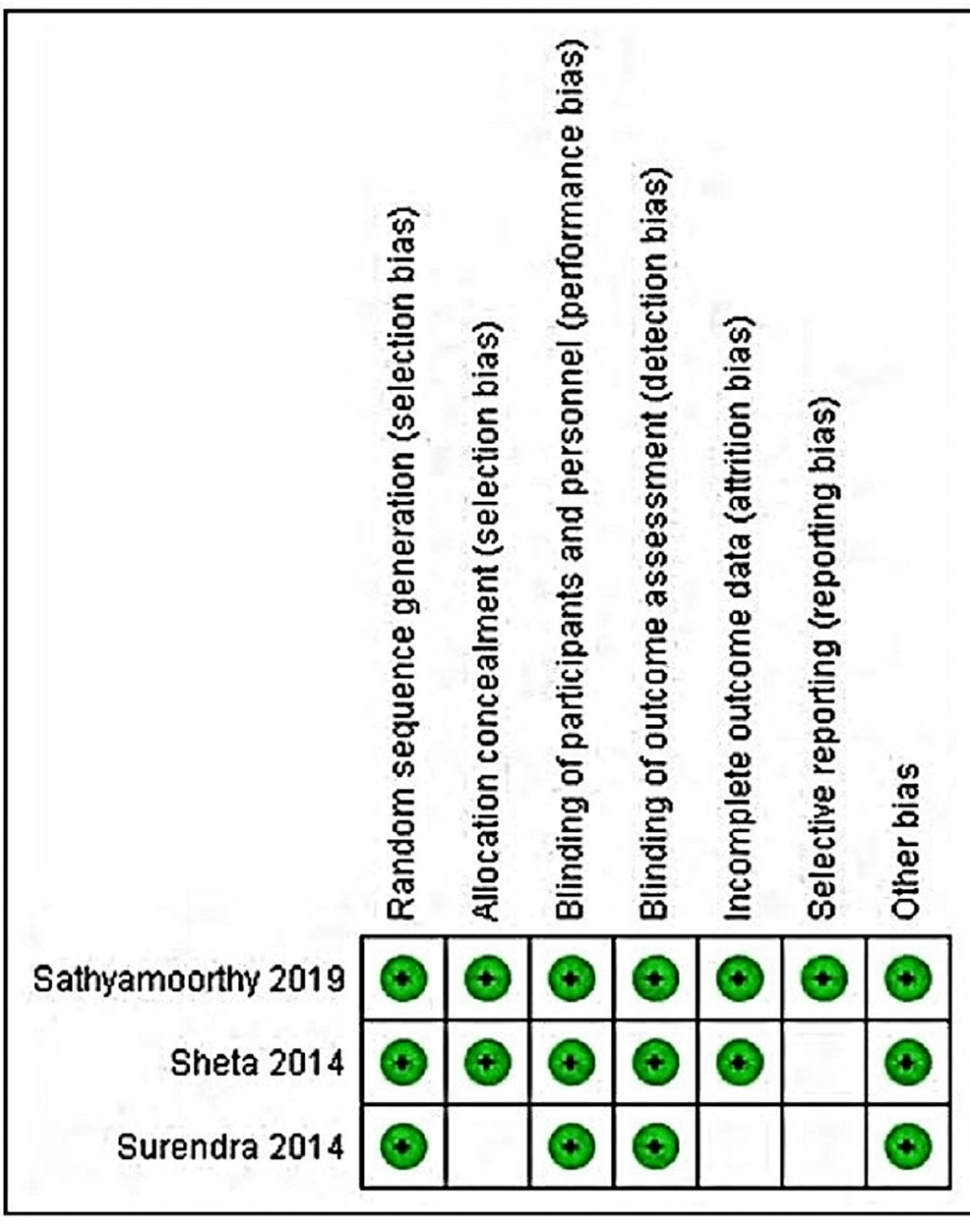
Risk of bias graph Reference [29-31]

Allocation: All of the three studies included in the review stated the use of either an online random sequence generator or a computer random sequence generator, which was followed throughout the study duration. Hence we addressed all three of them as low risk [29-31]. Concealment: Out of three, two of our studies used sealed envelopes for allocation [29,30]. Whereas another study did not clearly stated about the concealment of the allocation to the participants, and hence was kept unclear [31]. Blinding: One of the studies was single-blinded [30], whereas the rest two studies were double-blinded [29,31], due to which all three studies were kept in low-risk bias [29-31]. Incomplete outcome data: As the studies were completed in a single appointment, inherently due to the nature of the study, there were no cases of attrition in any of the studies [29-31]. Selective reporting: We categorized all three studies under low risk in the case of reporting. The performance or observational bias: In all the studies, all the procedures and observations were done by single trained calibrated personnel, hence we categorized the study as low risk in case of performance or observational bias. A summarization of the risk of bias in included studies and its graphical representation is given in Figure 2 and Figure 3, respectively.

Sensitivity analysis

We performed no sensitivity analyses. Regarding the missing data, assuming that these data were missing at random, we analyzed only the available data (i.e., ignoring the missing data).

Results

**Figure 4:**
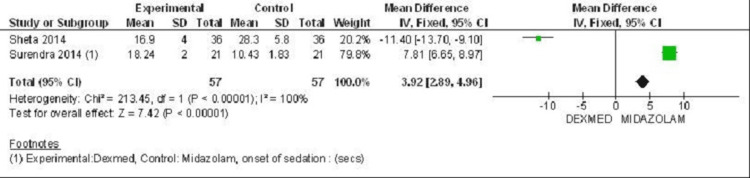
Forest plot 1 - Onset of sedation Reference [29,31]

Two studies were included for the parameter of onset of sedation [29,31]. Combined results showed heterogeneity of 100%. Hence, the effectiveness of dexmedetomidine and midazolam cannot be commented on. The Forest plot for the onset of sedation is shown in Figure 4.

**Figure 5:**
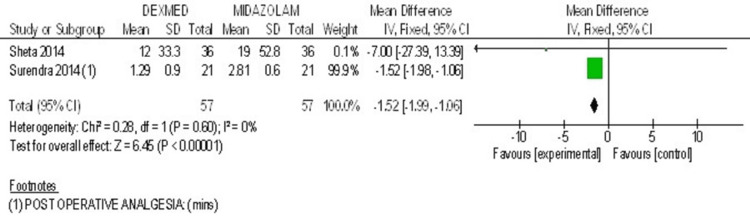
Forest plot 2 - Post operative analgesia Reference [29,31]

Two studies were included for the parameter of onset of sedation [29,31]. Combined results showed heterogeneity of 0%. It favored dexmedetomidine over midazolam. The Forest plot for postoperative analgesia is shown in Figure 5.

**Figure 6:**
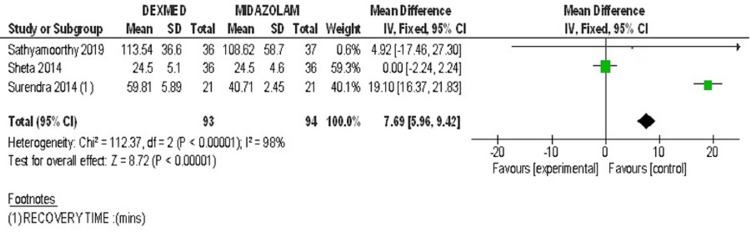
Forest plot 3 - Recovery time Reference [29-31]

Three studies were included for the parameter of recovery time [29-31]. Combined results showed heterogeneity of 100%. Effectiveness cannot be commented on. The Forest plot for the recovery time is shown in Figure 6.

Discussion

The sedative and anxiolytic drug midazolam can be given to kids in a variety of ways as a premedication. Each of these has particular advantages and disadvantages. However, intranasal delivery of midazolam has been contrasted in trials because of its ease and efficiency [29-31]. There is rising evidence to support the use of dexmedetomidine as an adjuvant to sedation and anesthesia in children [29]. Dexmedetomidine is a potent, highly selective, and specific a2 adrenoreceptor agonist that has sedative and analgesic properties. Its induced sedation can be identified by a smooth, quick waking from it that resembles sleep. Whether and how to take a premedication pill is still up for dispute. It is best to provide an intranasal sedative that is efficient, safe, and quick-acting. Most children consent to intranasal premedication either immediately or shortly after, with some persuasion from their mothers or with only a minimal amount of physical restraint [30]. When given intranasally to children, midazolam has been shown in the past to be a beneficial premedication agent [29]. However, employing intranasal midazolam premedication may be discouraged by the sensation of burning, nasal irritation, and a child's cries. In pediatric dentistry operations, these two sedatives are routinely used. However, there is not enough information to say which is better.

We included three studies reporting data from 229 participants aged below 3-18 years, comparing dexmedetomidine with midazolam in pediatric sedation. Studies reported data on the onset of sedation, depth of sedation, and recovery time. Overall completeness and applicability of evidence: An efficient, effective, and safe sedative could be used in routine general practice, this would benefit a wide range of patients, especially those who are phobic, anxious, and uncooperative by nature. This review focuses on answering which sedative agent is better to be used in pediatric populations. We analyzed three studies that met our decided inclusion criteria. After the assessment, although the bias in these studies was found to be low, we still state that the quality of evidence is low. This can be considered one of the limitations of this meta-analysis. As the number of clinical trials on this topic is less and we found only three RCTs to be included in this meta-analysis, this suggests that the data on this topic is not sufficient. Hence, through this review, we suggest that more RCTs should be carried out on this topic in the future.

## Conclusions

This review intended to answer whether dexmedetomidine or midazolam is a better sedative agent in pediatric dental practice. As we are aware that children in dental operatories are mostly fearful and anxious and those patients are managed by pediatric dentists most of the time by behavior management techniques. But those extremely uncooperative with dental treatment increase the distress and also affect the treatment outcomes. In such cases, pharmacological management of patients is indicated. Pharmacological methods such as sedation are resorted to in order to lower the agitation and anxiety of the child so that he/she is amicable to behavior management. We found three studies to include in our review. The preferred drug for its efficacy and in terms of patient satisfaction is dexmedetomidine. Despite the fact that dexmedetomidine is superior to midazolam in terms of effectiveness and dependability, an overall conclusion cannot be drawn because of limited RCTs on this topic. However, within the limitations of this review. We classified the evidence as low certainty. As the number of clinical trials on this topic is less and we found only three RCTs to be included in this meta-analysis, this suggests that the data on this topic is not sufficient. Hence, through this review we recommend more RCTs to be conducted on this subject matter in the future.
